# On the fractional Fourier and continuous fractional wave packet transforms of almost periodic functions

**DOI:** 10.1186/s13660-017-1402-3

**Published:** 2017-06-02

**Authors:** Banu Ünalmış Uzun

**Affiliations:** Department of Mathematics, Işık University, Şile, Istanbul 34980 Turkey

**Keywords:** 42A05, 42A38, 44A20, fractional Fourier transform, fractional wave packet transform, almost periodic function, frame

## Abstract

We state the fractional Fourier transform and the continuous fractional wave packet transform as ways for analyzing persistent signals such as almost periodic functions and strong limit power signals. We construct frame decompositions for almost periodic functions using these two transforms. Also a norm equality of this signal is given using the continuous fractional wave packet transform.

## Introduction

The fractional Fourier transform (FrFT), which is a generalization of the classical Fourier transform (FT), was introduced many years ago for solving the differential equation in quantum mechanics. Today, it is one of the most commonly used tools in signal processing. It has been investigated in numerous papers including [1–3]. Since it is potentially useful, it seems to have remained largely unknown in signal processing field. Also it has been introduced in optics as a fundamental tool for optical information processing [4, 5]. FrFT has opened up the possibility of useful applications including the use and detection of chirp signals, correlation and pattern recognition, space or time-variant filtering, Synthetic Aperture Radar (SAR) image processing, etc. [6, 7]. FrFT is likely to have something to offer in every area in which Fourier transform and related concepts are used. The well-known properties of FT have been extended to FrFT as in [8–10].

Besides FT, time-frequency representations of signals, such as Wigner distribution, short time Fourier transform (STFT)

$$S(\tau,f)=\frac{1}{\sqrt{2\pi }} \int_{-\infty }^{\infty } e^{-iut}g(t- \tau)f(t)\,dt, $$

where $g(t)$ is the window function, and wavelet transform (WT)

$$W(b,a)=\frac{1}{\sqrt{a}} \int_{-\infty }^{\infty } f(t) \overline{ \psi \biggl( \frac{t-b}{a} \biggr) }\,dt, $$

where *a* is the scaling and *b* is the translation parameters, are widely used linear transforms in speech processing, image processing or quantum physics.

1.1 $$ \psi_{b,a}(t)=\frac{1}{\sqrt{a}}\psi \biggl( \frac{t-b}{a} \biggr),\quad b\in \mathbb{R}, a>0 $$

is known as the mother wavelet.

The wave packet transform (WPT) [11] is the combination of STFT and continuous WT, that is the Fourier transform of a function windowed with a wavelet that is scaled by *a* and translated by *b*:

1.2 $$ WP(u,b,a)=\frac{1}{\sqrt{2\pi a}} \int_{-\infty }^{\infty }f(t) e ^{-iut} \overline{\psi \biggl( \frac{t-b}{a} \biggr) }\,dt. $$

Here, the function $\frac{1}{\sqrt{2\pi a}}e^{iut} \psi ( \frac{t-b}{a} ) $ is known as the wavelet packet.

Using the idea of FrFT, WPT and fractional mother wavelet $\psi_{b,a,\alpha }$, Huang and Suter [12] proposed the concept of the fractional wave packet transform (FrWPT), and Prasad et al. [13] modified FrWPT introducing the continuous fractional wave packet transform (CFrWPT).

In this paper, we firstly introduce FrFT and CFrWPT and some of their properties in brief. For a continuous signal $f(t)$, FrFT turns out to be a continuous-frequency function $F_{\alpha }(u)$, where *u* is the frequency associated with the fractional domain and *α* is the fraction. CFrWPT is related to FrFT since they both use the same kernel. We use these transforms as tools for analyzing almost periodic functions and strong limit power signals. Generalized frame decompositions for almost periodic functions are constructed by using FrFT and CFrWPT. We also give a version of norm identity for an almost periodic function connected with CFrWPT.

## Preliminaries

### The fractional Fourier transform

We define the Fourier transform of a function $f(t)$ as

$$F(u)=\frac{1}{\sqrt{2\pi }} \int_{-\infty }^{\infty }f(t)e^{-iut}\,dt $$

so that its inverse is

$$f(t)=\frac{1}{\sqrt{2\pi }} \int_{-\infty }^{\infty }F(u)e^{iut}\,du. $$

The fractional Fourier transform with angle *α* of a signal $f(t)$ is defined as

2.1 $$ F_{\alpha }(u)= { \int_{-\infty }^{\infty }} K_{\alpha }(t,u) f(t)\,dt, $$

where the kernel is

2.2 $$ K_{\alpha }(t,u)=\textstyle\begin{cases} \sqrt{\frac{1-i\cot \alpha }{2\pi }} e^{i\frac{t^{2}+u^{2}}{2} \cot \alpha - iut\csc \alpha }, & \alpha \ne n\pi, \\ \delta (t-u), & \alpha = 2n\pi, \\ \delta (t+u), & \alpha = (2n+1)\pi. \end{cases} $$

The original function $f(t)$ can be found by using the inverse FrFT of $F_{\alpha }$ as

2.3 $$ f(t)=\textstyle\begin{cases} \int_{-\infty }^{\infty } K_{-\alpha }(t,u) F_{\alpha }(u)\,du, & \alpha \ne n\pi, \\ F_{\alpha }(t), & \alpha = 2n\pi, \\ F_{\alpha }(-t), & \alpha = (2n+1)\pi, \end{cases} $$

where $K_{-\alpha }(t,u)=\sqrt{\frac{1+i\cot \alpha }{2\pi }} \exp \{ -i\frac{t^{2}+u^{2}}{2}\cot \alpha + iut\csc \alpha \} $.

When $\alpha =\pi /2$, FrFT reduces to the ordinary Fourier transform. For the new results, we deal with the case $\alpha \ne n\pi $.

In developing fractional Fourier series, we first find the orthogonal basis. Using inverse FrFT of an impulse function $\delta (t-nt_{0})$, we get

2.4 $$ \phi_{\alpha,n}(t)=\sqrt{\frac{1+i\cot \alpha }{2\pi }}\exp \biggl\{ -i\frac{t ^{2}+(nt_{0})^{2}}{2} \cot \alpha + i(nt_{0})t\csc \alpha \biggr\} , $$

where $t_{0}$ is called the central frequency in the fractional Fourier domains. We can divide each $\phi_{\alpha,n}$ by $\sqrt{T\csc \alpha /(2\pi)}$ in order to obtain an orthonormal basis

2.5 $$ \tilde{\phi }_{\alpha,n}(t)=\sqrt{\frac{\sin \alpha +i\cos \alpha }{T}}e^{-i((t^{2}+(n(2\pi /T)\sin \alpha)^{2})/2)\cot \alpha + int(2 \pi /T)}. $$

Thus, the fractional Fourier series expansion of a signal $f(t)$ can be written as

$$f(t)=\sum_{n=-\infty }^{\infty } C_{\alpha,n} \tilde{\phi }_{\alpha,n}(t) $$

on a finite interval, say $[-(T/2),(T/2)]$.

### The continuous fractional wave packet transform

The fractional wave packet transform of a function $f\in L^{2}( \mathbb{R})$ is defined as

2.6 $$ WP_{\alpha }(u,b,a)= \int_{-\infty }^{\infty }K_{\alpha }(t,u) \overline{ \psi_{b,a}(t)}f(t)\,dt, $$

where $K_{\alpha }(t,u)$ is the kernel defined in (2.2) and $\psi_{b,a}(t)$ is the mother wavelet defined in (1.1).

Shi et al. [14] gave the definition of fractional mother wavelet as

2.7 $$\begin{aligned} \psi_{b,a,\alpha }(t) =&\frac{1}{\sqrt{a}}\psi \biggl( \frac{t-b}{a} \biggr) e^{\frac{-i}{2}(t^{2}-b^{2})\cot \alpha } \\ =&\psi_{b,a}(t)e^{ \frac{-i}{2}(t^{2}-b^{2})\cot \alpha } \end{aligned}$$

for $b\in \mathbb{R}$, $a>0$ and any angle *α* which indicates the order. If we take $a=2^{-m}$ and $b=n2^{-m}$, we get $\psi_{m,n,\alpha }(t)=2^{m/2}\psi (2^{m} t-n) e^{\frac{-i}{2}(t^{2}-n ^{2} 2^{-2m})\cot \alpha }$ as the orthogonal set.

Using this fractional mother wavelet, the continuous fractional wave packet transform is given as

2.8 $$\begin{aligned}& \mathit{CWP}_{\alpha }(u,b,a) \\& \quad = \int_{-\infty }^{\infty }K_{\alpha }(t,u) \overline{ \psi_{b,a, \alpha }(t)}f(t)\,dt \\& \quad = \frac{C_{\alpha }}{\sqrt{a}} \int_{-\infty }^{\infty } e^{i\frac{t ^{2}+u^{2}}{2}\cot \alpha - iut\csc \alpha } e^{\frac{i}{2}(t^{2}-b ^{2})\cot \alpha } \overline{\psi \biggl( \frac{t-b}{a} \biggr) } f(t)\,dt, \end{aligned}$$

where $C_{\alpha }= \sqrt{\frac{1-i\cot \alpha }{2\pi }}$. Note that, for $\alpha =\pi /2$, CFrWPT corresponds to WPT.

### Almost periodic functions

We define by $L^{p}_{\mathrm{loc}}$ the space of the functions *f* on $\mathbb{R}$ such that the function $\Vert f\Vert ^{p}$ is locally Lebesgue integrable on $\mathbb{R}$ for $p\ge 1$. The space $\mathcal{AP}$ of almost periodic functions is the closure of quasi-periodic functions in $L^{p}_{\mathrm{loc}}$. This space consists of equivalence classes of functions of the form

$$f(t)= \sum_{k=1}^{\infty }a_{k} e^{i\lambda_{k}t}, $$

where $a_{k}\in \mathbb{C}$ and $\lambda_{k}\in \mathbb{R}$ (see [15, 16]). Equivalently, it is the completion of the space of trigonometric polynomials on $\mathbb{R}$ whose elements can be written as $\sum_{k=1}^{n} a_{k} e^{i\lambda_{k} t}$, where $n\in \mathbb{N}$, $a_{k}\in \mathbb{C}$ and $\lambda_{k}\in \mathbb{R}$. All $\mathcal{AP}$ functions are uniformly continuous and bounded, and we have

$$\Vert f\Vert ^{2}_{\mathcal{AP}}= \lim_{T\to \infty } \frac{1}{2T} \int _{-T}^{T} \bigl\vert f(t)\bigr\vert ^{2}\,dt. $$

Let $Q(\mathbb{R})$ consist of functions *q* in the form

2.9 $$ q(t)=\textstyle\begin{cases} \sum_{l=1}^{m} \lambda_{l} t^{\alpha_{l}}, & t\ge 0, \\ -\sum_{l=1}^{m} \lambda_{l} (-t)^{\alpha_{l}} ,& t< 0, \end{cases} $$

where $m=1,2,\ldots,\lambda_{l}\in \mathbb{R}$, $l=1,2,\ldots,m$ and $\alpha_{1}>\alpha_{2}>\cdots >\alpha_{m}>0$. A function of the form

$$P(t)=\sum_{k=1}^{n} a_{k} e^{iq_{k}(t)} $$

is called a generalized trigonometric polynomial on $\mathbb{R}$, where $a_{k}\in \mathbb{C}$, $q_{k}(t)\in Q(\mathbb{R})$, and $k=1,2,\ldots,n$. Denote by $\operatorname{Gtrig}(\mathbb{R})$ the set of all such polynomials. A function *f* on $\mathbb{R}$ is said to have strong limit power if for every $\varepsilon >0$ there exists $P_{\varepsilon }\in \operatorname{Gtrig}( \mathbb{R})$ such that

2.10 $$ \Vert f-P_{\varepsilon }\Vert =\sup \bigl\{ \bigl\vert f(t)-P_{\varepsilon }(t)\bigr\vert :t\in \mathbb{R}\bigr\} < \varepsilon. $$

Denote by $\mathcal{SLP}(\mathbb{R})$ the set of all such functions. The inner product of the $\mathcal{SLP}(\mathbb{R})$ space is defined by

$${\langle f,g\rangle }_{\mathcal{SLP}}= \lim_{T\to \infty } \frac{1}{2T} \int_{-T}^{T} f(t)\bar{g}(t)\,dt. $$

It is easy to see that $\mathcal{AP}(\mathbb{R})\subset \mathcal{SLP}( \mathbb{R})$ and $\Vert f\Vert _{\mathcal{AP}}=\Vert f\Vert _{\mathcal{SLP}}$ (see [17]). They both are the closed subspace of $L^{\infty }( \mathbb{R})$.

### The Parseval relation

The Parseval identity deals with the power of a function or a signal in the time and frequency domains. If $F(u)$ and $G(u)$ are the Fourier transforms of $f(t)$ and $g(t)$, respectively, we state the Parseval relation as

$$\int_{-\infty }^{\infty } f(t) \overline{g(t)}\,dt = \int_{-\infty } ^{\infty } F(u) \overline{G(u)}\,du. $$

The similar relation holds for the FrFT as

2.11 $$ \int_{-\infty }^{\infty } f(t) \overline{g(t)}\,dt = \int_{-\infty } ^{\infty } F_{\alpha }(u) \overline{G_{\alpha }(u)}\,du, $$

where $F_{\alpha }(u)$ and $G_{\alpha }(u)$ are the FrFT of $f(t)$ and $g(t)$ with order *α*, respectively [3]. If $f(t)=g(t)$, we get

2.12 $$ \int_{-\infty }^{\infty } \bigl\vert f(t)\bigr\vert ^{2}\,dt = \int_{-\infty }^{\infty } \bigl\vert F_{\alpha }(u)\bigr\vert ^{2}\,du. $$

If $f(t)$ is an $\mathcal{AP}$ function, it is easy to see that

2.13 $$ \bigl\Vert F_{\alpha }(u,\omega)\bigr\Vert _{L^{2}(u),\mathrm{AP}(\omega)} = \bigl\Vert f(t,\omega)\bigr\Vert _{L^{2}(t),\mathrm{AP}(\omega)}, $$

where

$$\bigl\Vert f(t,\omega)\bigr\Vert _{L^{2}(t),\mathrm{AP}(\omega)}=\lim_{T\to \infty } \frac{1}{2T} \int_{-T}^{T} \int_{-\infty }^{\infty }\bigl\vert f(t,\omega)\bigr\vert ^{2}\,dt \,d\omega. $$

## Main results

### Theorem 1

*Let* *f* *be an almost periodic function*. *And let* *α* *be an angle where* $\cot \alpha >0$ *and* $\alpha \ne n\pi $, $\alpha \ne \frac{(2n+1) \pi }{2}$, $n\in \mathbb{Z}$. *Then FrFT of* *f* *is a strong limit power function in* *u*.

### Proof

Let $f(t)=\sum_{k=1}^{n} a_{k} e^{i\lambda_{k} t}$ be a trigonometric polynomial. Then

3.1 $$\begin{aligned} &F_{\alpha }(u) \\ &\quad = { \int_{-\infty }^{\infty }} f(t)K_{\alpha }(t,u)\,dt \\ &\quad = C_{\alpha }\sum_{k=1}^{n} a_{k} \int_{-\infty }^{\infty } e^{i ( \lambda_{k}t+\frac{t^{2}}{2}\cot \alpha +\frac{u^{2}}{2}\cot \alpha -ut\csc \alpha) }\,dt \\ &\quad = C_{\alpha }\sum_{k=1}^{n} a_{k} e^{i \frac{u^{2}}{2}\cot \alpha } \int_{-\infty }^{\infty } e^{i [ (\lambda_{k}-u\csc \alpha)t+\frac{ \cot \alpha }{2}t^{2} ] }\,dt \\ &\quad = C_{\alpha } \sum_{k=1}^{n} a_{k} e^{i \frac{u^{2}}{2}\cot \alpha } \int_{-\infty }^{\infty } e^{\frac{i\cot \alpha }{2} [ ( t+\frac{ \lambda_{k}-u\csc \alpha }{\cot \alpha } ) ^{2}- ( \frac{ \lambda_{k}-u\csc \alpha }{\cot \alpha } ) ^{2} ] }\,dt \\ &\quad = C_{\alpha } \sum_{k=1}^{n} a_{k} e^{\frac{i\cot \alpha }{2} [ u ^{2}- ( \frac{\lambda_{k}-u\csc \alpha }{\cot \alpha } ) ^{2} ] } \int_{-\infty }^{\infty } e^{-\frac{\cot \alpha }{2i} ( t+\frac{ \lambda_{k}-u\csc \alpha }{\cot \alpha } ) ^{2}}\,dt, \end{aligned}$$

where $C_{\alpha }=\sqrt{\frac{1-i\cot \alpha }{2\pi }}$. Since

$$\int_{-\infty }^{\infty } e^{-\frac{\cot \alpha }{2i}u^{2}\,du}=\sqrt{ \frac{2 \pi i}{\cot \alpha }}, $$

we obtain

3.2 $$\begin{aligned} F_{\alpha }(u) &= \sqrt{1+i\tan \alpha } \sum_{k=1}^{n} a_{k} e^{\frac{i \cot \alpha }{2} [ u^{2}- ( \frac{\lambda_{k}-u\csc \alpha }{ \cot \alpha } ) ^{2} ] } \\ &= \sqrt{1+i\tan \alpha } \sum_{k=1}^{n} a_{k} e^{i [ (\cot \alpha -\csc 2\alpha)u^{2}+\lambda_{k}\sec \alpha u-\frac{\lambda ^{2}_{k} \cot \alpha }{2} ] }, \end{aligned}$$

which is a generalized trigonometric polynomial in *u*.

If *f* is a general almost periodic function, then there exists a sequence $(f_{n})$ of trigonometric polynomials where $f_{n}\to f$ uniformly. Since $F_{{\alpha }_{n}}(u)\in \operatorname{Gtrig}(\mathbb{R})$, we get $F_{\alpha }(u)\in \mathcal{SLP}(\mathbb{R})$.

Thus it is sufficient to verify that, if $\Vert f_{n} - f\Vert _{\infty } \to 0$, then $\Vert F_{{\alpha }_{n}}(u)-F_{\alpha }(u)\Vert _{L_{\infty }(y)} \to 0$.

Using the definition of FrFT, it is easy to see that

$$\bigl\vert F_{\alpha }(u)\bigr\vert \le \sqrt{\frac{1-i\cot \alpha }{2\pi }} \int_{- \infty }^{\infty }\bigl\vert f(t)\bigr\vert \,dt $$

and

3.3 $$ \bigl\vert F_{{\alpha }_{n}}(u)-F_{\alpha }(u)\bigr\vert \le \sqrt{ \frac{1-i\cot \alpha }{2\pi }} \int_{-\infty }^{\infty }\bigl\vert f_{n}(t)-f(t) \bigr\vert \,dt\to 0. $$

Since $\Vert F_{{\alpha }_{n}}(u)-F_{\alpha }(u)\Vert _{L_{\infty }(y)} = \sup \vert F_{{\alpha }_{n}}(u)-F_{\alpha }(u)\vert \to 0$, hence the result follows. □

### Theorem 2

*There exist constants* $A,B>0$ *such that*

3.4 $$\begin{aligned} A\Vert f\Vert ^{2}_{\mathcal{AP}} \le& \lim _{N\to \infty } \frac{1}{2N+1} \sum_{n=-N}^{N} \bigl\vert \langle f,\tilde{\phi }_{\alpha,n}\rangle \bigr\vert ^{2} \\ \le& B\Vert f\Vert ^{2}_{\mathcal{AP}} \end{aligned}$$

*for all almost periodic functions* *f*.

### Proof

Let us begin with the case when *f* is a trigonometric polynomial $f(t)=\sum_{k=1}^{K} a_{k} e^{i\lambda_{k} t}$. Since

$$\begin{aligned} \langle f, \tilde{\phi }_{\alpha,n} \rangle =&\sum_{k=1}^{K} a_{k} \sqrt{\frac{ \sin \alpha +i\cos \alpha }{T}} e^{\frac{i}{2} ( \frac{n2\pi }{T} \sin \alpha) ^{2}\cot \alpha } \int_{-\infty }^{\infty } e^{i\frac{t ^{2}}{2}\cot \alpha +i ( \lambda_{k}-n\frac{2\pi }{T} ) t}\,dt \\ = & \sum_{k=1}^{K} a_{k} \sqrt{ \biggl( \frac{2\pi i}{\cot \alpha } \biggr) \frac{ \sin \alpha +i\cos \alpha }{T}} e^{\frac{i}{2} ( \frac{n2\pi }{T} \sin \alpha) ^{2}\cot \alpha } e^{\frac{-i}{2\cot \alpha } ( \lambda _{k}-n\frac{2\pi }{T} ) ^{2}}, \end{aligned}$$

we calculate

3.5 $$\begin{aligned} &\frac{1}{2N+1} \sum _{n=-N}^{N} \bigl\vert \langle f, \tilde{\phi }_{\alpha,n} \rangle \bigr\vert ^{2} \\ & \quad =\frac{1}{2N+1}\sum_{n=-N}^{N} \sum _{k=1}^{K} \sum _{\ell =1}^{K} \frac{2 \pi }{T\cot \alpha }a_{k} \bar{a}_{\ell }e^{\frac{-i}{2\cot \alpha } ( \lambda_{k}-n\frac{2\pi }{T} ) ^{2}} e^{\frac{i}{2\cot \alpha } ( \lambda_{\ell }-n\frac{2\pi }{T} ) ^{2}} \\ &\quad =\frac{2\pi }{(2N+1)T\cot \alpha }\sum_{n=-N}^{N} \Biggl( \sum_{k=1} ^{K} \vert a_{k}\vert ^{2} + \sum {'} a_{k}\bar{a}_{\ell }j(\lambda_{k}, \lambda_{\ell }) \Biggr), \end{aligned}$$

where the last sum in (3.5) is taken over those *k*, *ℓ* such that $\lambda_{k}-\lambda_{\ell }$ is a (nonzero) multiple of $4\pi \cot \alpha $. In this case,

$$\begin{aligned} &\Bigl\vert \sum {'} a_{k} \bar{a}_{\ell }j(\lambda_{k},\lambda_{ \ell })\Bigr\vert \\ &\quad = \biggl\vert \sum_{\lambda \in \mathbb{R}} \sum _{s\in \mathbb{Z}\backslash \{0\}} a_{\lambda }\bar{a}_{\lambda +4 \pi s\cot \alpha } e^{\frac{-i}{2\cot \alpha } ( \lambda -n\frac{2 \pi }{T} ) ^{2}} e^{\frac{i}{2\cot \alpha } ( \lambda +4\pi s \cot \alpha -n\frac{2\pi }{T} ) ^{2}} \biggr\vert \\ &\quad \le \sum_{s\in \mathbb{Z}\backslash \{0\}} \biggl( \sum _{\lambda \in \mathbb{R}} \vert a_{\lambda }\vert ^{2} \bigl\vert e^{\frac{-i}{2\cot \alpha } ( \lambda -n\frac{2 \pi }{T} ) ^{2}}\bigr\vert \bigl\vert e^{\frac{i}{2\cot \alpha } ( \lambda +4 \pi s\cot \alpha -n\frac{2\pi }{T} ) ^{2}}\bigr\vert \biggr) ^{1/2} \\ &\qquad {} \times \biggl( \sum_{\lambda \in \mathbb{R}} \vert a_{\lambda +4\pi s\cot \alpha }\vert ^{2} \bigl\vert e^{\frac{-i}{2\cot \alpha } ( \lambda -n\frac{2 \pi }{T} ) ^{2}}\bigr\vert \bigl\vert e^{\frac{i}{2\cot \alpha } ( \lambda +4 \pi s\cot \alpha -n\frac{2\pi }{T} ) ^{2}}\bigr\vert \biggr) ^{1/2} \\ &\qquad \le \sum_{s\in \mathbb{Z}\backslash \{0\}} \sum _{\lambda \in \mathbb{R}} \vert a_{\lambda }\vert ^{2}. \end{aligned}$$

As $N \to \infty $, we get inequality (3.4) for the trigonometric polynomials. A standard approximation argument completes the proof for almost periodic functions. □

### Theorem 3

*Let* *f* *be an almost periodic function*, *α* *be an angle different from* *nπ*, *where* $n\in \mathbb{Z}$, *and let* $a=\sqrt{\pi }$. *Then CFrWPT of* *f* *is a strong limit power function in* *b*.

### Proof

Let $f(t)=\sum_{k=1}^{n} a_{k} e^{i\lambda_{k} t}$ be a trigonometric polynomial. Then

$$\begin{aligned} &\mathit{CWP}_{\alpha }(u,b,a) \\ &\quad =\frac{C_{\alpha }}{\sqrt{a}} \int_{-\infty }^{\infty } \sum_{k=1} ^{n} a_{k} e^{i\lambda_{k} t} e^{i\frac{t^{2}+u^{2}}{2}\cot \alpha - iut \csc \alpha } e^{\frac{i}{2}(t^{2}-b^{2})\cot \alpha }\overline{ \psi \biggl( \frac{t-b}{a} \biggr) }\,dt \\ &\quad = \frac{C_{\alpha }}{\sqrt{a}} e^{\frac{i}{2}(u^{2}-b^{2})\cot \alpha } \sum_{k=1}^{n} a_{k} \int_{-\infty }^{\infty } e^{i(\lambda _{k}-u\csc \alpha)t+i\cot \alpha t^{2}} \overline{\psi \biggl( \frac{t-b}{a} \biggr) }\,dt \\ &\quad = C_{\alpha }\sqrt{a} e^{\frac{i}{2}(u^{2}-b^{2})\cot \alpha } \sum_{k=1}^{n} a_{k} \int_{-\infty }^{\infty } e^{i(\lambda_{k}-u \csc \alpha)(va+b)+i\cot \alpha (va+b)^{2}} \overline{\psi (v)}\,dv \\ &\quad = C_{\alpha }\sqrt{a} e^{\frac{i}{2}(u^{2}-b^{2})\cot \alpha } \sum_{k=1}^{n} a_{k} e^{i(b\lambda_{k}-ub\csc \alpha +b^{2}\cot \alpha)} \\ &\qquad {} \cdot \int_{-\infty }^{\infty } e^{ia^{2}v^{2}\cot \alpha -i(2^{-1}ua-2^{-1} \lambda_{k} a\sin \alpha -ab\cos \alpha)2v\csc \alpha } \overline{ \psi (v)}\,dv, \end{aligned}$$

where we have made the substitution $v=(t-b)/a$. If we take $A=ua-\lambda_{k} a\sin \alpha -2ab\cos \alpha $ and $a=\sqrt{ \pi }$, we get

$$\begin{aligned} \mathit{CWP}_{\alpha }(u,b,\sqrt{\pi }) =& C_{\alpha }\pi^{1/4} e^{ \frac{i}{2}(u^{2}-b^{2})\cot \alpha } \sum_{k=1}^{n} a_{k} e^{i(b \lambda_{k}-ub\csc \alpha +b^{2}\cot \alpha)-i\pi {A^{2}}\cot \alpha } \\ &{} \cdot \int_{-\infty }^{\infty }e^{{i\pi (v^{2}+A^{2})}\cot \alpha -iAv \csc \alpha } \overline{\psi (v)}\,dv. \end{aligned}$$

Therefore,

3.6 $$ \mathit{CWP}_{\alpha }(u,b,\sqrt{\pi }) = \pi^{1/4} \sum _{k=1}^{n} a_{k} \Psi_{\alpha }(A) e^{iq_{k}(b)}, $$

where $\Psi_{\alpha }(A)$ is FrFT of mother wavelet *ψ* and

$$\begin{aligned} q_{k}(b) =& \biggl( \frac{1}{2}\cot \alpha -4\pi^{2} \cos^{3}\alpha \csc \alpha \biggr) b^{2} \\ &{} + \bigl[ \lambda_{k}-u\csc \alpha -(\lambda_{k}-u\csc \alpha)4\pi^{2} \cos^{2}\alpha \bigr] b \\ &{} + \biggl( \frac{1}{2}-\pi^{2} \biggr) {u^{2}}\cot \alpha -{\lambda_{k} ^{2}}\pi^{2}\sin \alpha \cos \alpha + 2{\lambda_{k}}\pi^{2} u\cos \alpha. \end{aligned}$$

Hence, we see that $\mathit{CWP}_{\alpha }(u,b,\sqrt{\pi })$ is a generalized trigonometric polynomial, and the result follows as in the proof of Theorem 1. □

### Theorem 4

*Let* *f* *be an almost periodic function and* *ψ* *be an* $L^{1}$ *admissible wavelet*. *Then*

3.7 $$ \bigl\Vert \mathit{CWP}_{\alpha }(u,b,\sqrt{\pi })\bigr\Vert _{L^{2}(u),AP(b)} = C_{\psi } \Vert f\Vert ^{2}_{AP}. $$

### Proof

Since $f(t)=\sum_{k=1}^{n} a_{k} e^{i\lambda_{k} t}$ is a trigonometric polynomial, we get

$$\bigl\Vert \mathit{CWP}_{\alpha }(u,b,\sqrt{\pi })\bigr\Vert _{AP(b)}= \frac{1}{2\sqrt{ \pi }} \sum_{k=1}^{n} \vert a_{k}\vert ^{2} \bigl\vert \Psi_{\alpha } \bigl( \sqrt{ \pi } ( u-\lambda_{k} \sin \alpha -2b\cos \alpha) \bigr) \bigr\vert ^{2}. $$

By using (2.11),

3.8 $$\begin{aligned} \bigl\Vert \mathit{CWP}_{\alpha }(u,b,\sqrt{\pi })\bigr\Vert _{L^{2}(u),AP(b)} &= \frac{1}{2\sqrt{ \pi }} \sum_{k=1}^{n} \int_{-\infty }^{\infty } \vert a_{k}\vert ^{2} \bigl\vert \psi (t)\bigr\vert ^{2}\,dt \\ &= C_{\psi } \Vert f\Vert ^{2}_{AP}. \end{aligned}$$

For general almost periodic functions *f*, we take a sequence $f_{n}$ such that $\Vert f_{n}-f\Vert _{\infty }\to 0$, and take them to be Bochner-Fejer approximants of the form $f_{n}(t)=\sum_{k=1}^{K_{n}}a _{k}^{(n)}e^{i\lambda_{k} t}$ (see [15]). Then

$$\begin{aligned} \bigl\Vert \mathit{CWP}_{\alpha }^{n}(u,b,\sqrt{\pi })-\mathit{CWP}_{\alpha }(u,b,\sqrt{\pi })\bigr\Vert _{L^{\infty }(b)}\le C_{\alpha }\sqrt{a} \Vert f_{n}-f\Vert _{\infty } \Vert \psi \Vert _{1}, \end{aligned}$$

using the monotone convergence theorem, the limiting argument of (3.8) will be valid, and the result follows. □

### Theorem 5

*There exist constants* $A,B>0$ *such that*

3.9 $$ A\Vert f\Vert ^{2}_{\mathcal{AP}} \le \sum _{m\in \mathbb{Z}} 2^{m} \lim_{N\to \infty } \frac{1}{2N+1} \sum_{n=-N}^{N} \bigl\vert \langle f,\psi_{m,n,\alpha }\rangle \bigr\vert ^{2} \le B \Vert f\Vert ^{2}_{\mathcal{AP}} $$

*for all almost periodic functions* *f*.

### Proof

If *f* is a trigonometric polynomial $f(t)=\sum_{k=1}^{K} a_{k} e^{i \lambda_{k} t}$, we see that

$$\begin{aligned} \langle f, \psi_{m,n,\alpha } \rangle =& 2^{m/2} \sum_{k=1}^{K} a _{k} \int_{-\infty }^{\infty } \overline{\psi \bigl(2^{m} t-n\bigr)} e^{ \frac{i}{2}(t^{2}-n^{2} 2^{-2m})\cot \alpha } e^{i\lambda_{k} t}\,dt \\ = & 2^{-m/2} \sum_{k=1}^{K} e^{i\frac{\lambda_{k}}{2^{m}}n} a_{k} \int_{-\infty }^{\infty } \overline{\psi (v)} e^{\frac{i}{2} ( \frac{v ^{2}}{2^{2m}}+\frac{2nv}{2^{2m}} ) \cot \alpha } e^{i\frac{\lambda _{k}}{2^{m}}v} \\ = & 2^{-m/2} \sum_{k=1}^{K} e^{i\frac{\lambda_{k}}{2^{m}}n} a_{k} \overline{ \hat{\psi } \biggl( \frac{\lambda_{k}}{2^{m}} \biggr) } e^{\frac{-i}{2} ( \frac{\lambda_{k}^{2}}{2^{4m}}+\frac{2n\lambda_{k}}{2^{3m}} ) \cot \alpha }. \end{aligned}$$

Hence we can write

3.10 $$\begin{aligned}& \lim_{N\to \infty } \frac{1}{2N+1} \sum_{n=-N}^{N} \sum _{m=-\infty }^{\infty }\bigl\vert \langle f, \psi_{m,n,\alpha } \rangle \bigr\vert ^{2} \\& \quad = 2^{-m} \sum_{k=1}^{K} \vert a_{k}\vert ^{2} \sum_{m=-\infty }^{\infty } \biggl\vert \hat{\psi } \biggl( \frac{\lambda_{k}}{2^{m}} \biggr) \biggr\vert ^{2} \\& \qquad {} + 2^{-m} \sum {'} a_{k} \bar{a}_{\ell }j(\lambda_{k},\lambda_{\ell }), \end{aligned}$$

where the last sum in (3.10) is taken over those *k*, *ℓ* such that $\lambda_{k}-\lambda_{\ell }$ is a (nonzero) multiple of $2^{m}$. In this case,

$$\begin{aligned}& \Bigl\vert \sum {'} a_{k} \bar{a}_{\ell }j(\lambda_{k},\lambda_{ \ell })\Bigr\vert \\& \quad = \Biggl\vert \sum_{\lambda \in \mathbb{R}} \sum _{s\in \mathbb{Z}\backslash \{0\}} a_{\lambda }\bar{a}_{\lambda + s2^{m}}\sum _{m=-\infty }^{\infty }\hat{\psi } \biggl( \frac{\lambda }{2^{m}} \biggr) \overline{\hat{\psi } \biggl( \frac{\lambda }{2^{m}}+s \biggr) } e^{isn} e ^{\frac{-i}{2} ( \frac{s(2\lambda +s2^{m})}{2^{3m}}+ \frac{2sn}{2^{2m}} ) \cot \alpha } \Biggr\vert \\& \quad \le \sum_{s\in \mathbb{Z}\backslash \{0\}} \Biggl( \sum _{m=-\infty }^{\infty }\sum_{\lambda \in \mathbb{R}} \vert a_{\lambda }\vert ^{2} \biggl\vert \hat{\psi } \biggl( \frac{\lambda }{2^{m}} \biggr) \biggr\vert \biggl\vert \hat{\psi } \biggl( \frac{ \lambda }{2^{m}}+s \biggr) \biggr\vert \Biggr) ^{1/2} \\& \qquad {} \times \Biggl( \sum_{m=-\infty }^{\infty }\sum _{\lambda \in \mathbb{R}} \vert a_{\lambda +s2^{m}}\vert ^{2} \biggl\vert \hat{\psi } \biggl( \frac{\lambda }{2^{m}} \biggr) \biggr\vert \biggl\vert \hat{\psi } \biggl( \frac{\lambda }{2^{m}}+s \biggr) \biggr\vert \Biggr) ^{1/2} \\& \quad \le \sum_{s\in \mathbb{Z}\backslash \{0\}} \Biggl( \sum _{m=-\infty }^{\infty }\sum_{\lambda \in \mathbb{R}} \vert a_{\lambda }\vert ^{2} \biggl\vert \hat{\psi } \biggl( \frac{\lambda }{2^{m}} \biggr) \biggr\vert \biggl\vert \hat{\psi } \biggl( \frac{ \lambda }{2^{m}}+s \biggr) \biggr\vert \Biggr) ^{1/2} \\& \qquad {} \times \Biggl( \sum_{m=-\infty }^{\infty }\sum _{\lambda \in \mathbb{R}} \vert a_{\lambda }\vert ^{2} \biggl\vert \hat{\psi } \biggl( \frac{\lambda }{2^{m}}-s \biggr) \biggr\vert \biggl\vert \hat{\psi } \biggl( \frac{\lambda }{2^{m}} \biggr) \biggr\vert \Biggr) ^{1/2} \\& \quad \le \sum_{\lambda \in \mathbb{R}} \vert a_{\lambda }\vert ^{2} \sum_{s\in \mathbb{Z}\backslash \{0\}} \bigl(\Gamma (s)\Gamma (-s)\bigr)^{1/2}, \end{aligned}$$

where $\Gamma (s)=\sup_{\lambda \in \mathbb{R}}\sum_{m\in \mathbb{Z}}\vert \hat{\psi } ( \frac{\lambda }{2^{m}} ) \vert \vert \hat{\psi } ( \frac{\lambda }{2^{m}}+s) \vert $. If we assume

$$\begin{aligned}& A = \inf_{\lambda \in \mathbb{R}}\sum_{m\in \mathbb{Z}} \biggl\vert \hat{\psi } \biggl( \frac{\lambda }{2^{m}} \biggr) \biggr\vert ^{2}- \sum_{s\in \mathbb{Z}\backslash \{0\}} \bigl( \Gamma (s)\Gamma (-s) \bigr) ^{1/2}>0, \\& B = \sup_{\lambda \in \mathbb{R}}\sum_{m\in \mathbb{Z}} \biggl\vert \hat{\psi } \biggl( \frac{\lambda }{2^{m}} \biggr) \biggr\vert ^{2}+ \sum_{s\in \mathbb{Z}\backslash \{0\}} \bigl( \Gamma (s)\Gamma (-s) \bigr) ^{1/2}< \infty, \end{aligned}$$

we get inequality (3.9) for the trigonometric polynomials. Then we find the result for almost periodic functions by a standard approximation. □

## Conclusion

We analyzed the fractional Fourier transform and the continuous fractional wave packet transform for almost periodic signals. We construct frame decompositions for almost periodic functions using these two transforms. Also a norm equality of this signal is given using the continuous fractional wave packet transform.
